# UHV deposition and characterization of a mononuclear iron(III) β-diketonate complex on Au(111)

**DOI:** 10.3762/bjnano.5.223

**Published:** 2014-11-18

**Authors:** Irene Cimatti, Silviya Ninova, Valeria Lanzilotto, Luigi Malavolti, Luca Rigamonti, Brunetto Cortigiani, Matteo Mannini, Elena Magnano, Federica Bondino, Federico Totti, Andrea Cornia, Roberta Sessoli

**Affiliations:** 1 Laboratory of Molecular Magnetism, Department of Chemistry "Ugo Schiff", University of Florence & INSTM RU of Florence, Via della Lastruccia 3, 50019 Sesto Fiorentino, Italy; 2 Department of Chemical and Geological Sciences, University of Modena and Reggio Emilia & INSTM RU of Modena and Reggio Emilia, Via G. Campi 183, 41125 Modena, Italy,; 3 CNR-IOM, Laboratorio TASC, Basovizza SS-14, Km 163.5, 34149 Trieste, Italy

**Keywords:** Au(111), β-diketonate complexes, DFT, STM, thin films, UPS, XMCD, XPS

## Abstract

The adsorption of the sterically hindered β-diketonate complex Fe(dpm)_3_, where Hdpm = dipivaloylmethane, on Au(111) was investigated by ultraviolet photoelectron spectroscopy (UPS) and scanning tunnelling microscopy (STM). The high volatility of the molecule limited the growth of the film to a few monolayers. While UPS evidenced the presence of the β-diketonate ligands on the surface, the integrity of the molecule on the surface could not be assessed. The low temperature STM images were more informative and at submonolayer coverage they showed the presence of regular domains characterized by a flat morphology and height of ≈0.3 nm. Along with these domains, tetra-lobed features adsorbed on the kinks of the herringbone were also observed. DFT-simulated images of the pristine molecule and its possible decomposition products allowed to assess the partial fragmentation of Fe(dpm)_3_ upon adsorption on the Au(111) surface. Structural features with intact molecules were only observed for the saturation coverage. An ex situ prepared thick film of the complex was also investigated by X-ray magnetic circular dichroism (XMCD) and features typical of high-spin iron(III) in octahedral environment were observed.

## Introduction

A renewed interest in mononuclear metal complexes has recently arisen due to the observation that systems of this class can behave as single molecule magnets (SMMs) [1–6]. SMMs are molecules whose magnetic moment reorients orders of magnitude slower than in normal paramagnets and results in a memory effect at low temperature. Such a behaviour is often accompanied by spectacular quantum features, for example, resonant quantum tunnelling of the magnetization [7–9], and has attracted practical interest in the areas of ultra-high-density information storage devices, quantum computing and spintronics [10]. Although the SMM behaviour was first observed in polynuclear systems, the investigation was extended to simple mononuclear complexes of either lanthanide or transition-metal ions, which are better suited for vapour-phase processing, in particular when β-diketonate ligands are present [11].

This work exploits the high volatility of the iron(III) tris-β-diketonate complex, Fe(dpm)_3_ (Hdpm = dipivaloylmethane), in order to perform a detailed in situ ultra-high vacuum (UHV) characterization. In Fe(dpm)_3_ the three dipivaloylmethanide ligands chelate a high-spin (HS) Fe^3+^ ion, producing a distorted octahedral coordination environment. Fe(dpm)_3_ is of specific importance because in a previous study it was suggested as a possible contaminant in thin films of [Fe_4_(Ph-C(CH_2_O)_3_)_2_(dpm)_6_] (Fe_4_Ph) [12], a tetrairon(III) star-shaped SMM that can be sublimated in vacuum conditions. This class of molecules provided the first evidence that SMMs can retain their memory effect once grafted onto a metallic substrate. The magnetic properties of individual Fe_4_ molecules have also been addressed using electro-migrated nanojunctions [13–16]. We present here a detailed scanning tunneling microscopy (STM) and photoelectron spectroscopy investigation, in the ultraviolet (UPS) and X-ray (XPS) ranges, on ultra-thin films of Fe(dpm)_3_ sublimated on Au(111) surfaces. The non-trivial interpretation of the STM images and the spectroscopic data, supported by theoretical simulations, evidence a pronounced reactivity of this species with gold surface.

## Results and Discussion

### Electronic characterization

**Figure 1:**
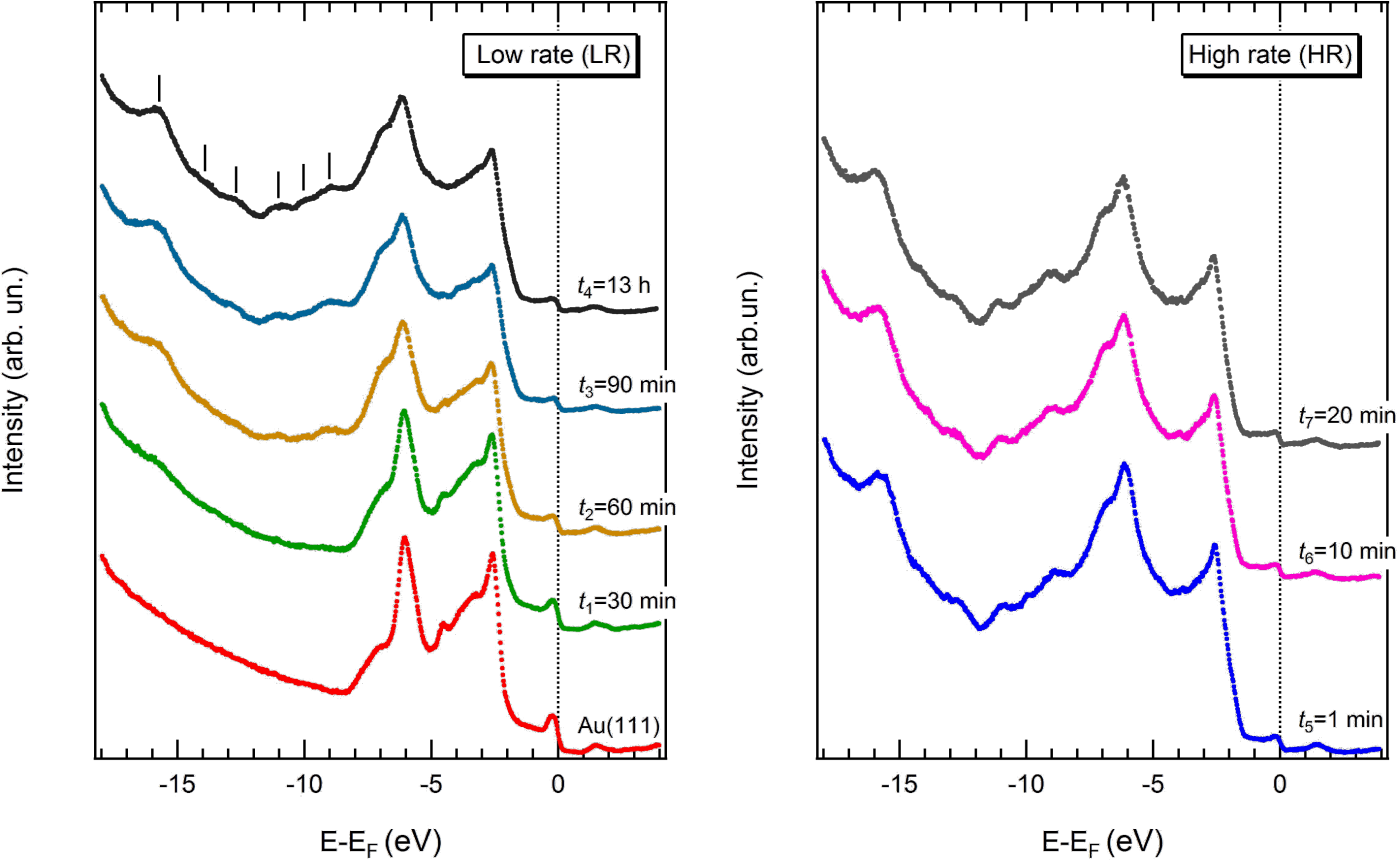
UPS spectra acquired for the Au(111) sample exposed to increasing doses of Fe(dpm)_3_ with low (left) and high (right) deposition rates.

The Fe(dpm)_3_ adsorption mechanism onto the Au(111) surface was studied by means of UPS and XPS measurements. Due to the high volatility of the compound, low deposition rates (LR) were obtained by keeping the crucible at room temperature and varying the exposure time, namely, *t*_1_ = 30 min, *t*_2_ = 60 min, *t*_3_ = 90 min, *t*_4_ = 13 h. The corresponding UPS sequence is reported in the left panel of Figure 1.

The spectrum recorded at *t*_1_ is almost identical to the one collected for the clean substrate. Only a slight attenuation of the gold features and the appearance of a small peak near −15.7 eV can be noticed. Longer exposure times (*t*_2_ and *t*_3_) lead to a clear development of the deeper molecular states and a more evident smearing of the gold valence band (VB). Finally, the spectrum shape of the sample dosed for ca. 13 h remains practically unchanged if compared with the *t*_3_ deposition. This result suggests a self-limiting adsorption mechanism of Fe(dpm)_3_ on the Au(111) surface. With the aim of collecting more material, the deposition rate (high rate, HR) was increased by heating the crucible up to 338 K. In this case, relatively short exposure times (*t*_5_ = 1 min) already show the typical features observed for the *t*_4_ sample (compare the right and left panel of Figure 1). Despite the high deposition rate, longer doses (*t*_6_ = 10 min and *t*_7_ = 20 min) do not produce thicker films, which confirms that saturation of the coverage has been achieved. This behaviour is fully consistent with that reported for other metal β-diketonate complexes. Saturation coverage has been observed for Cu(hfac)_2_, adsorbed onto the TiO_2_(110) substrate [17], while multilayers of Pd(hfac)_2_ can be obtained by cooling Cu surfaces at 120 K [18].

**Figure 2:**
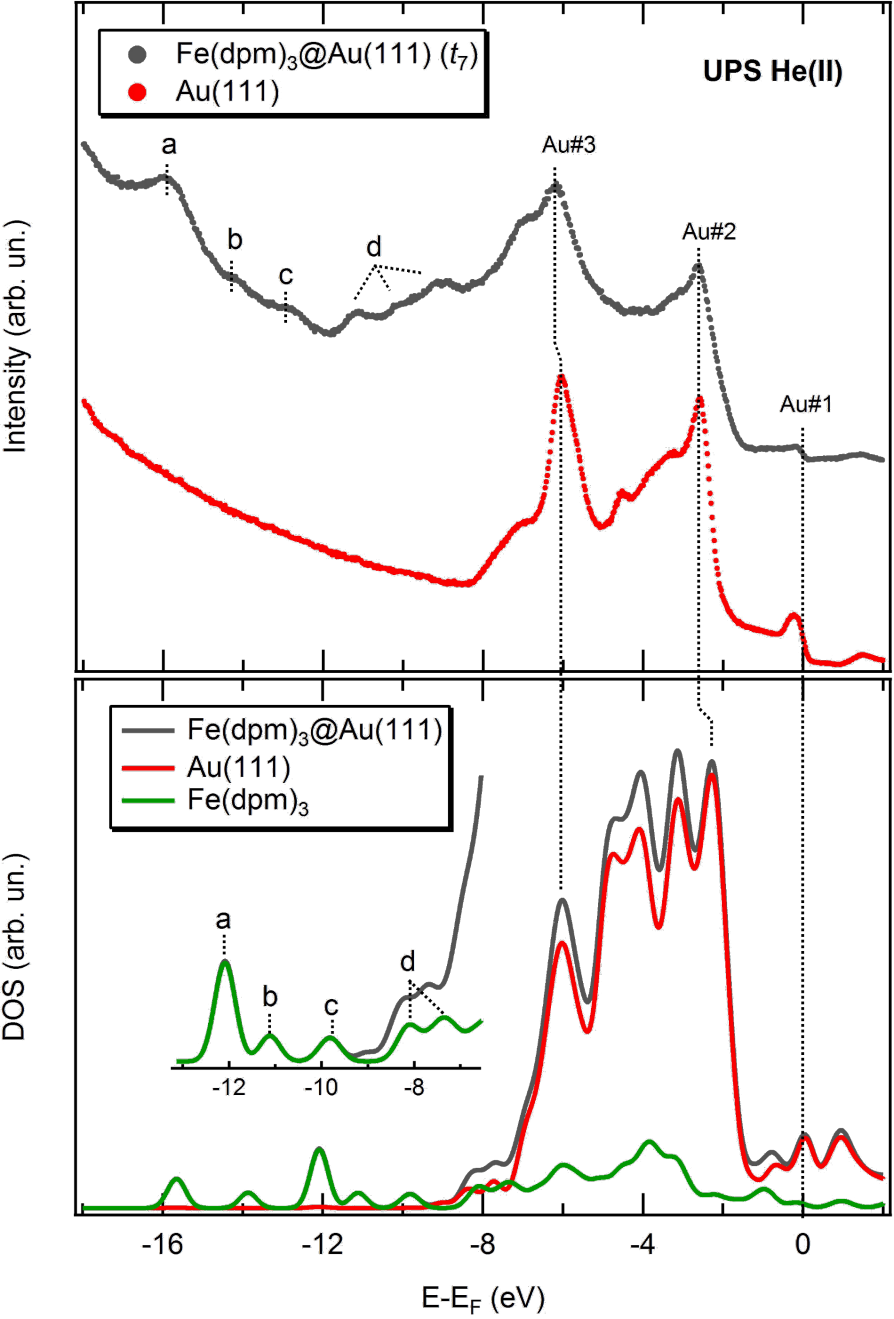
(Top panel) UPS spectra relative to the Fe(dpm)_3_ saturation coverage (grey curve) and the clean substrate (red curve). (Bottom panel) Theoretical density of states for the system Fe(dpm)_3_@Au(111) (grey curve) and decomposition into Au and Fe(dpm)_3_ contributions.

As shown in Figure 2 (top panel), the spectrum corresponding to the saturation coverage (*t*_7_) still displays some features related to the gold substrate; in particular, the Fermi edge (Au#1) and the most prominent peaks (Au#2 and Au#3) of the spectra between −2 and −7 eV can be clearly identified. On the other hand, the smooth trend of the inelastic electron tail allows observation of the molecular features labelled as a, b, c and d. To gain a deeper insight, the density of states (DOS) for the Fe(dpm)_3_@Au(111) system was computed through a periodic density functional approach (see details in Experimental section). The comparison between the experimental and computed DOS spectra (Figure 2) shows a good correlation between the main features. The DOS region between −2 and −7 eV is strongly dominated by the gold features while few molecular states are clearly visible only at higher binding energies, that is, at more negative values of E − E_F_ (see inset in the bottom panel of Figure 2). These deeper molecular states can be easily associated to those observed in the inelastic tail of the experimental spectrum, despite the contraction of the theoretical energy scale. The observed slight mismatch between experimental and theoretical energy scale can be related to possible deficiencies in the used exchange-correlation functional/basis sets combination [19]. However, it should be considered that the calculated DOS do not take into account that during the photoexcitation process the creation of a hole reduces the electron screening, the so-called final state effects in photoemission [20]. This effect becomes larger with a deeper created hole, justifying the larger discrepancies observed at higher binding energies.

**Figure 3:**
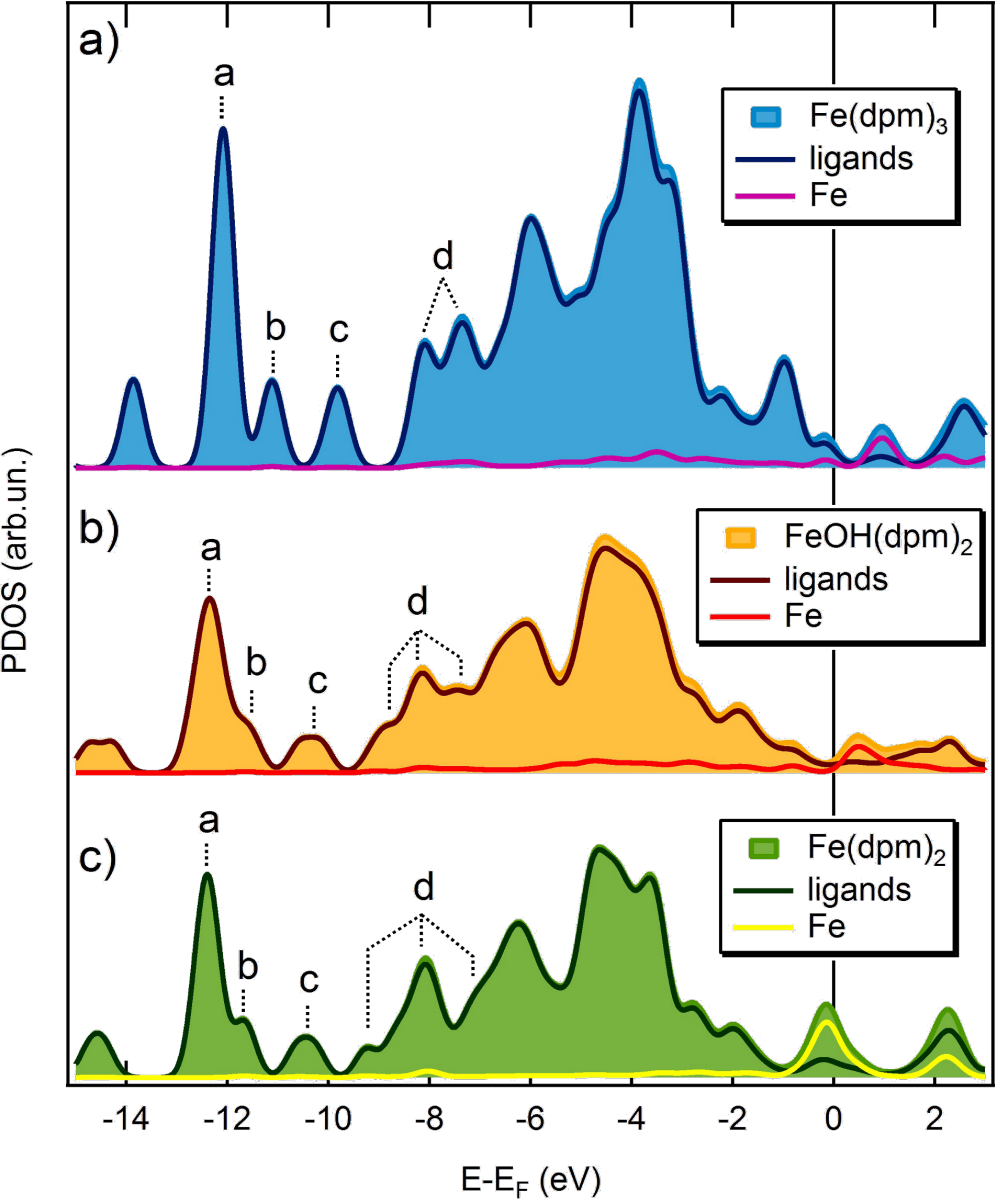
Projected DOS of the molecules in Fe(dpm)_3_@Au(111) (a), FeOH(dpm)_2_@Au(111) (b), and Fe(dpm)_2_@Au(111) (c), in which further separation of PDOS coming from the Fe ion and the ligands is presented.

By plotting the projected density of states (PDOS) on the ligands and the iron ion (see Figure 3a), it is evident that dpm^−^ ligands provide the main orbital contributions to the energy region where the molecular peaks (a, b, c, d) can be identified at the UPS level. More information on the coordination environment of the iron ion could be extracted from the frontier molecular orbitals which are also expected to bear the fingerprint of any possible molecule–substrate interaction. However, at low molecular coverage, the UPS spectra are dominated by the gold signal and no information on the molecule’s Fermi region could be obtained. Therefore, it seems that UPS spectra are unable to unambiguously assess the integrity of Fe(dpm)_3_ once adsorbed on the gold surface.

**Figure 4:**
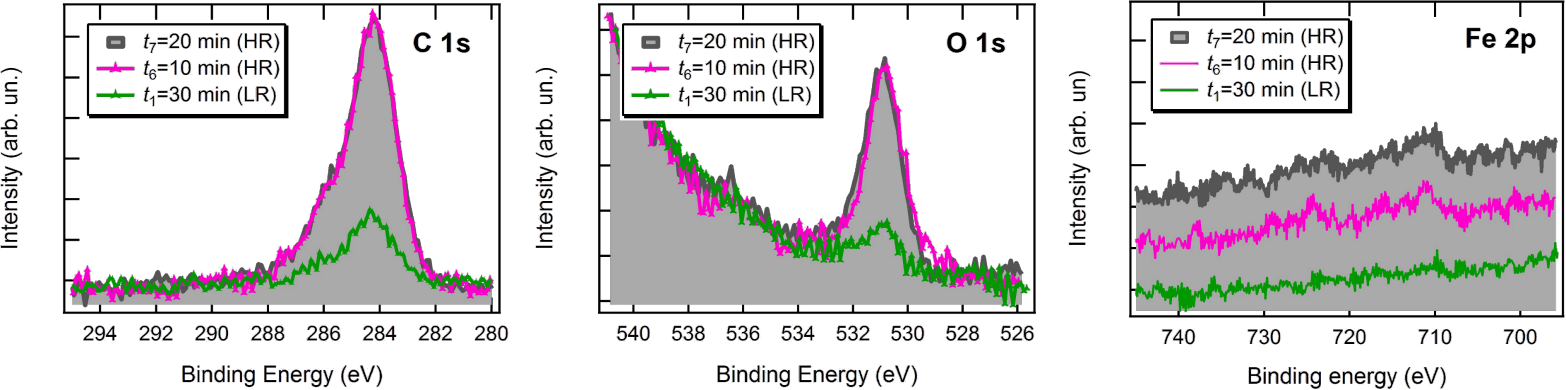
C 1s, O 1s and Fe 2p XPS spectra for the Au(111) substrate exposed to increasing doses of Fe(dpm)_3_.

Samples labelled as *t*_1_, *t*_6_, and *t*_7_ were also characterized by XPS spectroscopy, and the results are reported in Figure 4. As expected, no intensity variations occur when passing from *t*_6_ to *t*_7_. For the *t*_1_ coverage, lower than the saturation one, the C 1s and O 1s peaks do not show significant changes in terms of line shape and binding energy with respect to the thicker films. As for the Fe 2p region, the signal is detectable but quite noisy at saturation coverage, and practically negligible at *t*_1_. Therefore, no useful information about the Fe oxidation state could be retrieved.

### STM and DFT characterization

Spectroscopic characterization indicates that Fe(dpm)_3_ adsorbs on the gold surface up to a saturation coverage, probably one or two layers, but no definitive conclusions could be drawn about the molecule–substrate interaction. With the aim of identifing the nature of the deposited film, samples with saturation and submonolayer coverage were studied by means of low temperature STM measurements.

**Figure 5:**
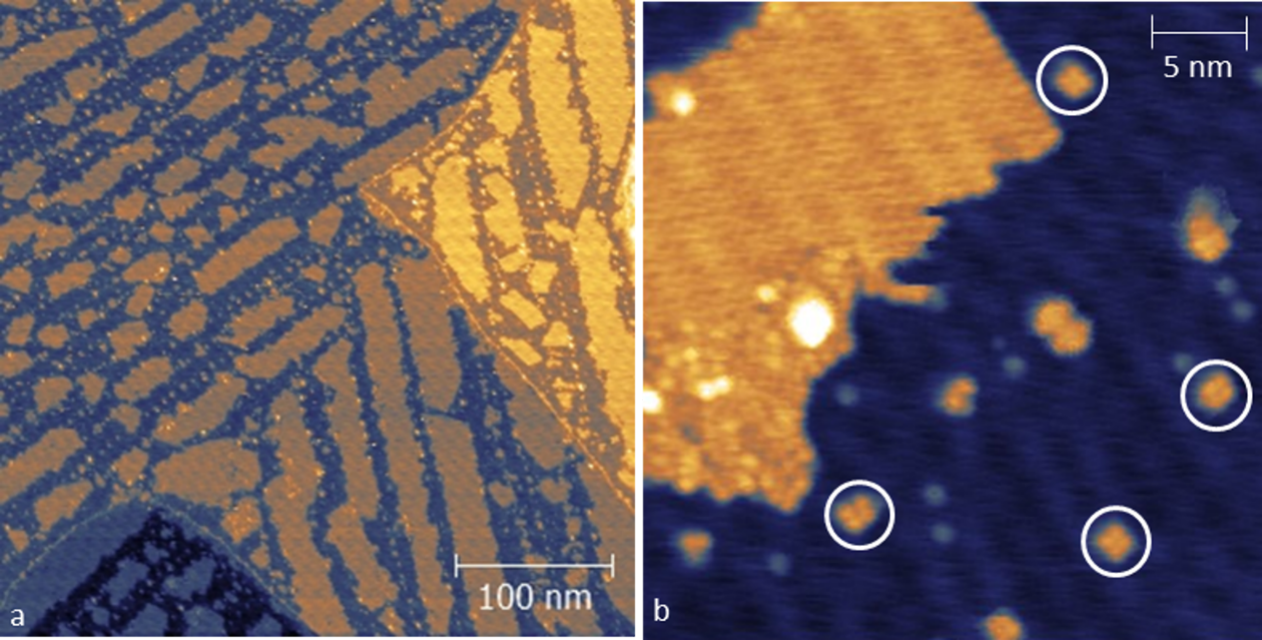
STM image of Au(111) surface after exposure to Fe(dpm)_3_ for *t*_1_ = 30 min (LR) at *T* = 30 K. (a) Size = 400 × 400 nm^2^, bias = −2 V (empty states), *I* = 10 pA. (b) Size = 34 × 34 nm^2^, bias = −2 V (empty states), *I* = 5 pA.

A representative STM image (400 × 400 nm^2^) for submonolayer coverage (*t*_1_ = 30 min) is presented in Figure 5a. Upon adsorption, the surface is characterized by the presence of molecular patches with regular shape and variable size. Reactive sites on the Au substrate, such as the kinks of the herringbone reconstruction and terrace steps, seem to be necessary for the nucleation of molecular domains. Extended islands can grow in the middle of a gold terrace starting from the isolated objects initially adsorbed on the herringbone kinks. Molecular assembly can also occur starting from the lower side of step edges. An enlarged view of the surface reveals that the molecular islands are mainly characterized by a flat morphology and an ordered internal structure (see Figure 5b). However, given the limited resolution, it is not possible to address the individual units forming these domains. On the other hand, their flatness and ordering suggest that these features could arise from the self-assembly of highly symmetric building blocks. Indeed, most of the isolated objects, which are believed to be the starting point of the molecular self-assembly, are characterized by a four-fold symmetry (indicated with open circles in Figure 5b). Moreover, both islands and isolated objects are 0.29 ± 0.02 nm high, therefore confirming the common nature of their building blocks. The ordered domains present also less ordered portions (see the bottom part of the island in Figure 5b). In some areas of the sample, a second layer is also observed and has the same ordered domains in addition to sporadic disordered dendritic regions. By comparing the height of ordered and disordered regions (both at the submonolayer and second layer domains) we can conclude that they might be constituted of the same units (see Figure S1 in Supporting Information File 1).

**Figure 6:**
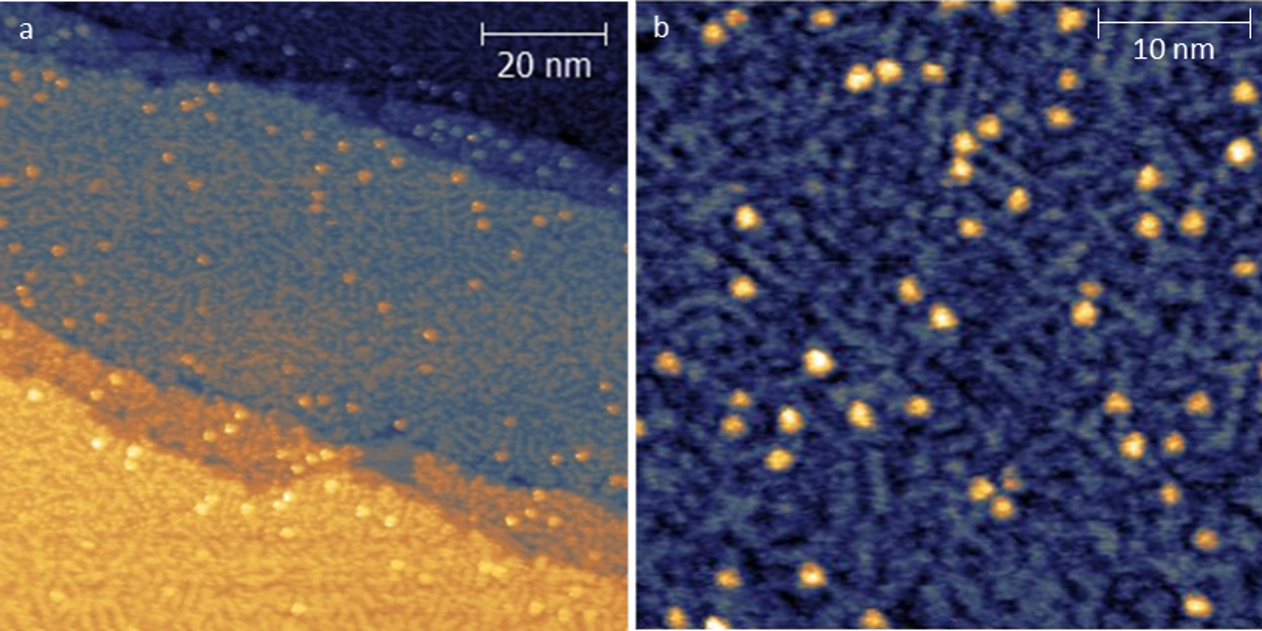
STM images for saturation coverage of Fe(dpm)_3_ on Au(111) at *T* = 30 K. (a) *t*_6_ = 10 min (HR); size = 100 × 100 nm^2^, bias = 1.5 V (filled states), *I* = 10 pA. (b) *t*_7_ = 1 min (HR); size = 45 × 45 nm^2^, bias = 1.5 V (filled states), *I* = 10 pA.

The situation is different for STM images corresponding to the saturation coverage, that is, *t*_6_ and *t*_7_ (see Figure 6). Both samples are characterized by a wetting layer whose dendritic morphology is reminiscent of the second layer disordered regions, which were occasionally detected at the submonolayer regime. This finding suggests that high deposition rates prevent the molecules from self-assembling in ordered domains. On top of this disordered layer we also observed quasi-spherical objects with a height of 0.35 ± 0.06 nm and a diameter of 1.57 ± 0.21 nm.

**Figure 7:**
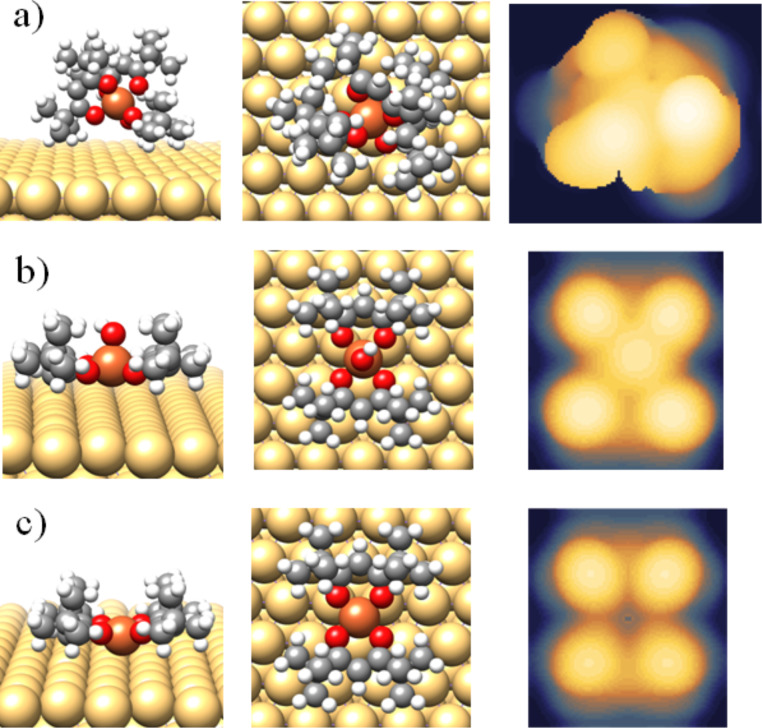
Optimized geometries of the three theoretical models Fe(dpm)_3_@Au(111) (a), FeOH(dpm)_2_@Au(111) (b), and Fe(dpm)_2_@Au(111) (c) presented as side (left column) and top views (middle column). Simulated STM images at experimental bias are also reported in the right column. (a) Bias = −1.5 eV (filled states); size = 17.31 × 14.99 Å^2^. (b), (c) Bias = 2 eV (empty states); size = 14.44 × 14.99 Å^2^.

To get a deeper insight in the adsorption process, the STM image of Fe(dpm)_3_ was simulated by DFT calculations. At the experimental bias of 1.5 V (negative values for simulations), an almost spherical multi-lobe image with height of about 0.92 nm and diameter of approximately 1.37 nm is calculated (see Figure 7a). A reasonable agreement between the calculated image and the round features of Figure 6 was found. However, the limited experimental resolution and the approximation in the calculation approach do not allow for an unambiguous conclusion. Because of the low resolution, it is much more difficult to find correlations with the features observed within the dendritic layer or other disordered regions.

As for the submonolayer coverage (Figure 5b), the observed tetra-lobed features (and probably the flat domains) are not compatible with the spherical calculated aspect for intact Fe(dpm)_3_ molecules, suggesting that major structural changes occur on the gold substrate, namely, decomposition. To better understand the features observed at low coverage deposition, two molecular fragments were theoretically investigated as possible intermediate or end products in the Fe(dpm)_3_ decomposition process: FeOH(dpm)_2_@Au(111) and Fe(dpm)_2_@Au(111). Indeed, the high-spin (HS) Fe^3+^ ion in FeOH(dpm)_2_ can undergo reduction to HS Fe^2+^ in Fe(dpm)_2_ via Fe(d_z_^2^)–Au(s) interaction and result in low-spin (LS) Fe^2+^ with a concurrent release of the OH^−^ group. FeOH(dpm)_2_ considers a penta-coordinated complex of HS Fe^3+^ with two dpm^−^ ligands forming the basis of a square pyramid and the OH^−^ group acting as an apical ligand. Fe(dpm)_2_ corresponds to a LS Fe^2+^ square planar complex. The optimized geometries as well as the computed STM images are reported in Figure 7b,c. The computed STM image of Fe(dpm)_2_@Au(111) (Figure 7c) matches very closely to the observed tetra-lobed units with no detectable contribution from the iron d_z_^2^ orbital. FeOH(dpm)_2_@Au(111) also affords a tetra-lobed pattern, but with an extra spot in the middle. This shows that FeOH(dpm)_2_ is unlikely to be the end product of Fe(dpm)_3_ decomposition.

The TDOS and PDOS for the two fragments were also computed and compared to the ones of pristine Fe(dpm)_3_ in Figure 3. The largest differences are expected in the valence band region involving the coordination site (i.e., molecules Fermi region). Unfortunately, as mentioned above, these features are hidden by the gold contribution. Even if some minor differences are computed for the inner levels corresponding to the dpm^−^ ligands, again the overwhelming contribution from the substrate does not allow for unambiguous identification of the species present on the surface from UPS experiments.

Thanks to the combined STM and DFT investigation we partially rationalized the adsorption mechanism of Fe(dpm)_3_ on the Au(111) surface in terms of a "dissociative adsorption process". This is also supported by the exhaustive literature which can be found on the surface reactivity of metal β-diketonates in relation with their use as metallic precursors in coating technology, such as chemical vapour deposition (CVD) and atomic layer deposition (ALD) [21–22]. For instance, the reactivity of Cu^II^(hfac)_2_, hfac^−^ = hexafluoroacetylacetonate, was found to critically depend on the nature of the molecule–substrate interaction. Using TiO_2_(110) [17], Ag [23], TiN [24–25], and Ta [26] as substrates, the molecule dissociatively chemisorbs giving rise to “activated” species (Cu^I^hfac and hfac^−^) which favour the subsequent reduction to Cu^0^ by chemical processing [23,25] or thermal treatment [17,26]. On the contrary, Cu(hfac)_2_ adsorbs on SiO_2_ without fragmentation, thus making reduction to Cu metal less favoured [27]. In the case of Cr(dbm)_3_, dbm^−^ = dibenzoylmethanide, the STM investigation revealed bi-lobed features associated with free dbm^–^, suggesting that the molecule dissociatively interacts with the Cu(100) surface, while the less reactive dbm-based Ru complexes seem to adsorb as intact molecules on Ag(111) [28–29].

A different situation is observed for complexes based on Fe(II) and bearing pyridine ligands, such as Fe((H_2_B-pz)_2_)_2_(bipy), Fe((H_2_B-pz)_2_)_2_(phen) or Fe(phen)_2_(NCS)_2_, where H_2_B-pz = bis(hydrido)bis(1*H*-pyrazol-1-yl)borate, bipy = 2,2’-bypiridine and phen = 1,10-phenanthroline. This class of compounds, known as spin crossover (SCO) [30], can be reversibly switched between two distinct spin states, low-spin (LS) and high-spin (HS), by means of a variety of external inputs, such as temperature, light and charge flow. Recently many efforts have been made to study SCO molecules adsorbed on solid surfaces with the aim to exploit their conversion properties in nanoscale devices [31–36]. Many of these studies have systematically shown the presence of intact molecules even if the switching properties can be dramatically altered by the interaction of the organic ligands with the surface. For instance, the electrical switching of Fe((H_2_B-pz)_2_)_2_(phen) can be observed in the second molecular layer deposited on Au(111), but the molecules of the first layer cannot be switched [36]. Similarly, isolated Fe(phen)_2_(NCS)_2_ molecules cannot be switched on Cu(100). On the other hand, the introduction of an interfacial layer of CuN on Cu(100) allows switching between the HS and the LS state [33,35]. A slightly different situation was observed for a submonolayer of Fe((H_2_B-pz)_2_)_2_(bipy) on Au(111) [34], where 20% of the molecules are able to preserve the SCO behavior.

### XMCD of a Fe(dpm)_3_ thick film

**Figure 8:**
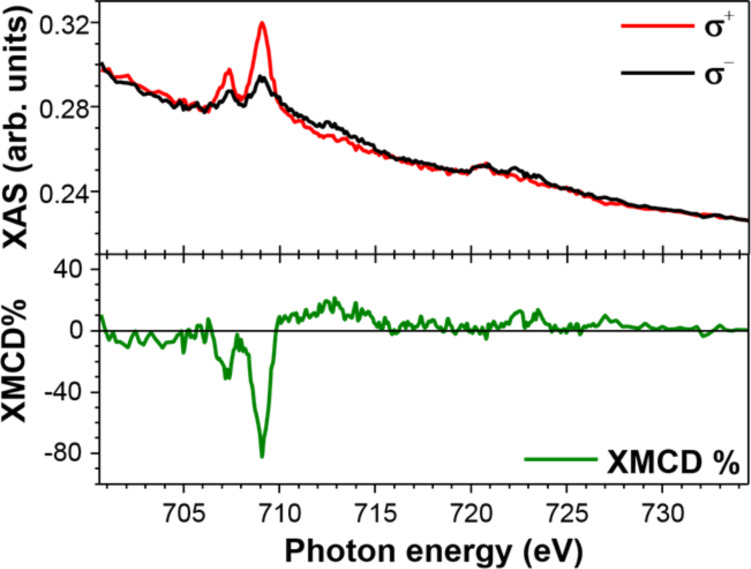
X-ray absorption spectra for a bulk sample of Fe(dpm)_3_ acquired using the left (σ^+^) and right (σ^−^) circular polarisation (upper panel) and the derived XMCD% spectrum calculated by dividing the XMCD signal (σ^−^ − σ^+^) by the L_3_ edge jump of the isotropic spectrum (σ^−^ + σ^+^)/2 (lower panel).

Given the interest in Fe(dpm)_3_ as a potential contaminant of evaporable Fe_4_ SMMs [12], the magnetic characterization of an ex situ preparation was also attempted. Considering that the high coverages compatible with an ex situ prepared sample cannot be achieved by UHV sublimation, a thick film sample of Fe(dpm)_3_ was prepared by drop-casting. XAS spectra at the Fe L_2,3_ edge, acquired at the BACH beamline of the Elettra synchrotron for both circular left (σ^+^) and circular right (σ^−^) polarization, are reported in the top panel of Figure 8. These absorption spectra were measured at 4 K under an external field of 3 T applied parallel to the light propagation vector. They show the expected features for HS Fe^3+^ ions in octahedral coordination geometry with two distinct peaks at the L_3_ edge [37–39]. From these data the XMCD signal can be extracted as the difference (σ^−^ − σ^+^).

Similarities between the presented XMCD spectra, featuring the largest intensity at 709.1 eV, and those reported for the Fe_4_ family of molecules [13–14,40] are evident thus excluding radiation damage for Fe(dpm)_3_ molecules in the adopted experimental conditions. The amplitude of the XMCD% signal reaches approximately 80% of the isotropic contribution (σ^−^ + σ^+^)/2, as expected for a set of independent HS Fe^3+^ ions with their magnetic moment fully aligned in the direction of the externally applied magnetic field [37–38]. Interestingly this value is comparable to the one recorded at the Fe L_2,3_ edge on the heteronuclear Fe_3_Cr systems [41], the isostructural chromium centred analogues of Fe_4_ SMMs. On the other hand, the XMCD% intensity observed here is almost twice as large as in Fe_4_ SMMs. We reiterate here that in star-shaped Fe_4_ SMMs, the field-opposing contribution of the central spin halves the average magnetic polarization per iron site. Full polarization is instead achieved in these conditions for non-interacting Fe^3+^ ions, as in the present case, or for the peripheral and parallel aligned Fe^3+^ spins of Fe_3_Cr.

It is interesting to point out that also the XMCD profile observed in Fe(dpm)_3_ is very close to that of Fe_3_Cr. In particular, for both Fe(dpm)_3_ and Fe_3_Cr, the XMCD signal remains negative in the saddle between the two main peaks at the L_3_ edge (707.9 eV). By contrast, the XMCD signal at 707.9 eV vanishes in Fe_4_ SMMs [13–14,40]. The different behaviour of the latter can be justified by a non-perfect cancellation of the magnetic contribution of central and peripheral Fe^3+^ ions, thus confirming that this spectral feature is a diagnostic signal for intact star-shaped Fe_4_ molecules [41].

## Conclusion

Our multi-technique investigation revealed, notwithstanding from the sterically hindered β-diketonate ligands, Fe(dpm)_3_ undergoes a partial decomposition upon adsorption on the Au(111) surface. The high volatility of the complex limits the deposition to only a few layers. Photoelectron spectroscopy of valence and core states proved to be unable to assess the presence of intact complexes on the surface. More informative was an in situ, low temperature STM investigation, which showed the presence of both tetra-lobed and approximately spherical objects, the latter only visible for higher coverages on top of a wetting layer. The comparison of the experimental topography with DFT-simulated STM images of the pristine Fe(dpm)_3_ complex, as well as those of two possible fragments, suggests that the observed tetra-lobed features are compatible with the formation of Fe(dpm)_2_ species on the surface, while the spherical spots visible at higher coverages reveal some resemblance with the simulated images for Fe(dpm)_3_. Despite the important information obtained by combining STM microscopy and DFT calculations, a definitive assessment of decomposition products in terms of redox and spin state could only be achieved through a detailed synchrotron investigation on in situ prepared samples.

## Experimental

### Synthesis of [Fe(dpm)_3_]

A solution of Hdpm (160.2 mg, 0.8693 mmol) in acetonitrile (5 mL) and NEt_3_ (0.4 mL) was added dropwise to a solution of sublimed FeCl_3_ (48.0 mg, 0.296 mmol) in acetonitrile (1 mL). A red, microcrystalline solid was formed and was collected and washed with acetonitrile (2 mL) and dried in vacuum (113.7 mg, 64.79%). Stoichiometric calculations for C_33_H_57_FeO_6_ (605.66) were: C, 65.44; H, 9.49, while experimental values revealed C, 65.01; H, 9.66. NMR studies revealed: ^1^H NMR (200 MHz, C_6_D_6_, 293 K, δ): 12.9 ppm (54 H, *t-*Bu) with mp 171–172 ºC. The unit cell of the crystals was checked by X-ray diffraction and found to coincide with that reported in the literature [42].

### Sample preparation

All UHV-based depositions were performed on a Au(111) single crystal. The surface was cleaned by repeated Ar^+^ sputtering (2 µA, 1 keV) and annealing (720 K) cycles. Considering that Fe(dpm)_3_ and most β-diketonates show high volatility [43–44], the sublimation was performed in a dedicated preparation chamber with a base pressure of 1 × 10^−7^ mbar. Low deposition rates were obtained by keeping the molecular powders, hosted in a quartz crucible, at room temperature. In order to achieve higher deposition rates, the powders were heated to a temperature of about 338 K. During the sublimation, the substrate was kept a room temperature. A K-type thermocouple, buried into the powder, allowed for temperature control.

### STM studies

The STM images were obtained by an UHV scanning tunnelling microscope (Omicron VT-STM) operating at 30 K in the constant current mode with electrochemically-etched W tips. The applied tip bias voltage and the tunnelling current of each image are given in the figure caption.

### Photoelectron spectroscopy

XPS and UPS measurements were carried out in an UHV chamber with a base pressure in the low 10^−10^ mbar range. The chamber is equipped with a hemispherical analyser (VSW HA100) with a 16-channel detector, a monochromatic X-ray source (Al Kα source, *E* = 1486.6 eV), and a helium discharge lamp. The X-ray source was assembled at 54.44º with respect to the analyser and operated at a power of 100 W (13 kV and 7.7 mA). For the UPS spectra, the He II line (40.8 eV) was used for excitation. In order to ensure that all photoelectrons generated by the He II line were detected, a fixed bias of −30 V was applied to the sample. Both XPS and UPS spectra were recorded in normal emission with circular 5 mm entrance and exit slits. The pass energy was set to 44 and 10 eV for XPS and UPS spectra, respectively. For the XPS spectra, the binding energy scale was calibrated by setting the Au 4f_7/2_ peak at 80.04 eV. UPS spectra were calibrated such that the Fermi level was located at 0 eV.

### X-ray absorption spectroscopy

The deposition was prepared by drop-casting using a 2 mM dichloromethane solution on a gold film grown on mica.

The Fe L_2,3_ XMCD measurements were performed in total electron yield using a ±6.5 Tesla, 2 K cryomagnet endstation at the BACH beamline of the Elettra synchrotron facility in Trieste (Italy) [45]. For the measurements we used magnetic fields of ±3 T applied in the same direction of the synchrotron light propagation, sample temperature of 4 K, energy resolution below 100 meV and theoretical 100% degree of circular polarization. In order to suppress beam damage, the flux was reduced to have sample drain currents below 11 pA. The data were normalized using a Au grid located between the sample and the last focusing mirror of the beamline.

### DFT calculations

The calculations for all model structures were performed with the Cp2k program package [46] within the DFT framework. The Grimme’s D3 parameterization approach [47] was used to introduce the dispersion correction term. Norm-conserving Goedecker–Teter–Hutter (GTH) pseudopotentials [48] were used together with GTH double-ζ polarized molecularly optimized basis sets for all atomic species. The energy cut-off applied to the plane wave basis sets was set to 400 Ry. Geometry optimizations were performed with the PBEsol functional [49]. In all cases, the convergence criteria were fixed at 1 × 10^−6^ Hartree for the SCF energy and 1 × 10^−3^ Hartree Bohr^−1^ for the atomic forces. A Fermi–Dirac distribution was used with a broadening (electronic temperature) of 300 K.

The following simulation cells sizes were used: Fe(dpm)_3_@Au(111) – (17.3 × 15.0 × 40.0) Å^3^ FeOH(dpm)_2_@Au(111) and Fe(dpm)_2_@Au(111) – (14.4 × 15.0 × 40.0) Å^3^. During the geometry optimization, the atomic positions of the bottom Au layer were kept fixed to the bulk experimental distances (2.885 Å), whereas the other two layers were allowed to relax. In all simulated DOS studies, the Gaussian width of the convolution, σ, was set to 0.30 eV. The STM images were simulated according to the Tersoff–Hamman approximation [50] as implemented in Cp2k.

## Supporting Information

Supporting information contains STM images of low rate deposition of Fe(dpm)3. An additional second layer of ordered and disordered domains is visible.

File 1 Additional STM images.
