# Supplementary material for: UHV deposition and characterization of a mononuclear iron(III) β-diketonate complex on Au(111)
Source: Beilstein J Nanotechnol. 2014 Nov 18;5:2139–48. doi: 10.3762/bjnano.5.223 (PMC4273306; doi:10.3762/bjnano.5.223)
Supplement: File 1 — Additional STM images. [file Beilstein_J_Nanotechnol-05-2139-s001.pdf]

Supporting Information

for

UHV deposition and characterization of a mononuclear iron(III) β-diketonate complex on Au(111)

Irene Cimatti[1], Silviya Ninova[1], Valeria Lanzilotto[1], Luigi Malavolti[1], Luca Rigamonti[2], Brunetto Cortigiani[1], Matteo Mannini[1], Elena Magnano[3], Federica Bondino[3], Federico Totti[1], Andrea Cornia[2] and Roberta Sessoli*[1]

Address: [1]Laboratory of Molecular Magnetism, Department of Chemistry Ugo Schiff, University of Florence & INSTM RU of Florence, Via della Lastruccia 3, 50019 Sesto Fiorentino, Italy, [2]Department of Chemical and Geological Sciences, University of Modena and Reggio Emilia & INSTM RU of Modena and Reggio Emilia, Via G. Campi 183, 41125 Modena, Italy, and [3]CNR-IOM, Laboratorio TASC, Basovizza SS-14, Km 163.5, 34149 Trieste, Italy

Email: Roberta Sessoli* - roberta.sessoli@unifi.it

* Corresponding author

Additional STM images

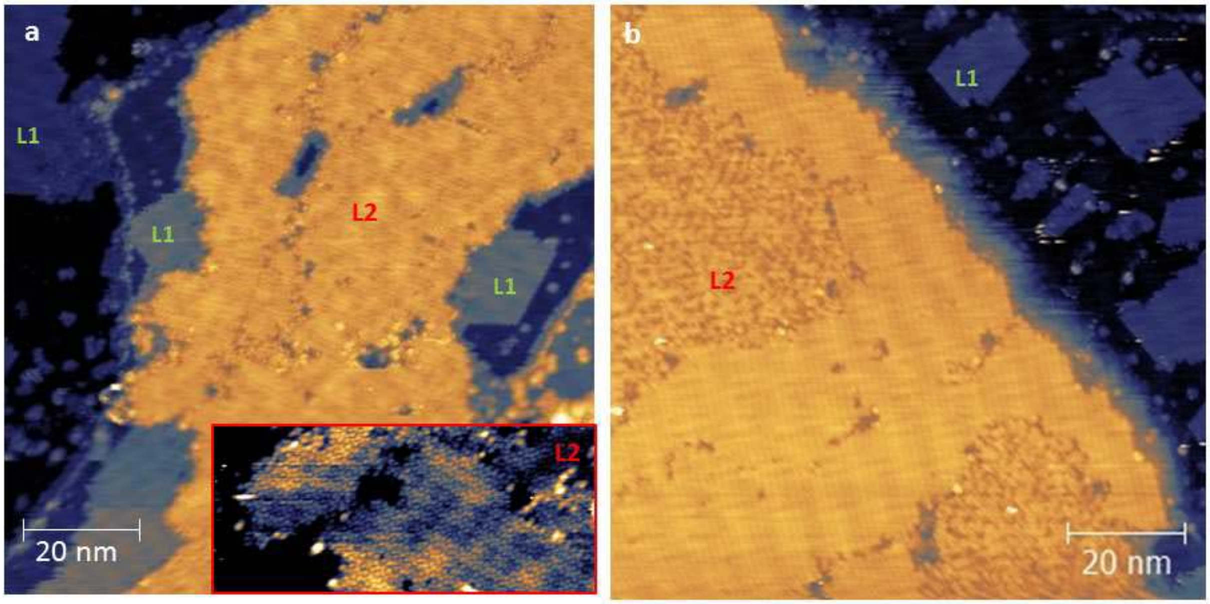

Figure S1: STM images of the Au(111) surface after exposure to $Fe(dpm)_3$ for t_1 = 30 min (low rate). First and second layer are indicated as L1 and L2, respectively. (a) Size = 100 × 100 nm^2, Bias = −2 V, I = 3 pA. (Inset) Enlarged view of L2, Size = 18 × 41 nm^2. (b) Size = 100 × 100 nm^2, Bias = −2 V, I = 5 pA. The height of L2 is 0.27 ± 0.04 nm, therefore comparable to L1 (0.29 ± 0.2 nm). Figure 1b shows a second layer characterized by ordered and disordered domains.
